# U-shaped association between hemoglobin to red blood cell distribution width ratio and all-cause mortality among critically ill pediatric patients

**DOI:** 10.1016/j.jped.2025.101491

**Published:** 2025-12-17

**Authors:** Weichao He, Jie Liu, Rui Jiang, Xinyu Yang, Xujie Zhang, Ruoyu Cao, Zhenshui Hu, Xiaolan Zhang, Yan Gao

**Affiliations:** a Cangzhou Central Hospital, Department of Pediatrics, Cangzhou, Hebei Province, China; b Chinese PLA General Hospital, Department of Vascular and Endovascular Surgery, Beijing, China; c Cangzhou Central Hospital, Department of Neonatology, Cangzhou, Hebei Province, China; d Blood Transfusion Research Laboratory, Cangzhou Central Blood Station, Cangzhou, Hebei Province, China; e Chinese Medicine Hall, Cangzhou Central Hospital, Cangzhou, Hebei Province, China; f Third People's Hospital of Liaocheng, Department of Pediatrics, Liaocheng, Shandong Province, China; g The 960th Hospital of PLA, Department of General Medicine, JiNan, ShanDong Province, China

**Keywords:** Hemoglobin and red blood cell distribution width (HRR), Mortality, Cohort study, Pediatric Intensive Care Unit (PIC) database

## Abstract

**Objective:**

The lower hemoglobin to red blood cell distribution width ratio (HRR) is associated with an increased risk of mortality in adult patients, but its relationship with clinical progress in the Pediatric Intensive Care Unit (PICU) is unclear. The authors aimed to investigate the association between HRR and all-cause mortality among pediatric patients.

**Methods:**

The authors conducted a retrospective cohort study by analyzing the PIC database from 2010 to 2018.HRR was calculated based on laboratory tests conducted within the first 24 hours of PICU admission. The primary outcome was 28-day in-hospital all-cause mortality. Multivariable logistic regression models, restricted cubic spline, and threshold effects analysis were applied to assess the relationship between HRR and mortality in this cohort.

**Results:**

A total of 8015 patients with an average age of the participants was1.5 (0.4, 4.8) years, and 3547 (44.3%) individuals were female. The 28-day in-hospital all-cause mortality was 4.1% (330/8015). The relationship between HRR and mortality was U-shaped, which had a threshold of around 8.91. The effect size on the left and right sides of the inflection point, was 0.803 (95% CI 0.742-0.869, p < 0.001) and 1.421 (95% CI 1.159-1.743, p < 0.001), respectively. No significant interactions were observed between HRR and all-cause mortality, except in patients with high lactate (p for interaction > 0.05). The results of the sensitivity analysis remained stable.

**Conclusions:**

There is a U-shaped relationship between HRR and 28-day in-hospital all-cause mortality in critically ill pediatric. With a lower mortality risk at an HRR of 8.91.

## Introduction

Mortality prediction and risk assessment are critical in public health and clinical practice.[1] Despite significant reductions in child mortality rates from 1990 to 2019 due to initiatives like the Sustainable Development Goals.[2] Pediatric mortality remains a global challenge, with poor quantification of risk in critically ill children. Reliable biomarkers are urgently needed to predict adverse outcomes, improve risk stratification, guide clinical decision-making, and optimize resource allocation.

Hemoglobin (Hb) is a crucial component of the complete blood count, closely related to nutritional status and immune response.[3,4] Research has demonstrated that low Hb levels have been linked to non-small cell lung cancer,[5] head and neck cancer,[6] colorectal cancer,[7] acute coronary syndrome,[8] ischemic stroke,[9] and non-traumatic intracerebral hemorrhage.[10] In addition, red cell distribution width (RDW) reflects the heterogeneity of red blood cell volume and has recently been recognized as an independent risk factor for mortality in the general population.[11] Elevated RDW levels have been associated with increased mortality in various patient populations, including those with cardiovascular and cerebrovascular disease,[12] community-acquired pneumonia,[13] cancer,[14,15] liver or kidney failure,[16] and sepsis,[17] as well as in other acute or chronic conditions. The hemoglobin-to-RDW ratio (HRR) reflects systemic inflammation and overall health status. However, both HRR levels may be influenced by various factors, such as medications, nutritional status, oxidative stress, and blood transfusion.[11,18]

Existing scoring systems like Pediatric Risk of Mortality (PRISM) and the Pediatric Index of Mortality (PIM) have limitations due to the extensive number of clinical variables required.[19,20] Recently, the HRR has been recognized as a novel composite marker reflecting oxygen-carrying capacity and erythropoietic stress. The HRR has been identified as an accurate and innovative prognostic indicator in several malignancies, with low HRR levels associated with poor outcomes in affected patients.[21,22] Nevertheless, the prognostic value of HRR concerning all-cause mortality in pediatrics remains scarce. To fill this knowledge gap, the present study aimed to investigate the association between HHR and 28-day all-cause mortality in critically ill pediatric patients.

## Materials and methods

### Data sources

This analysis utilized deidentified clinical records from the Pediatric Intensive Care Database (PIC v1.1) containing 13,941 critical care admissions (12,881 unique patients aged 0-18 years) at Children's Hospital, Zhejiang University School of Medicine from 2010-2018. The institutional review board granted exemption (Hangzhou, China 2019_IRB_052) [23] as data were anonymized and publicly accessible. Patient consent was waived due to privacy protection measures involving randomized coding. The first author, Weichao He, successfully completed the Collaborative Institutional Training Initiative (CITI) course and passed the "Conflicts of Interest" and "Data or Specimens Only Research" modules (ID: 41878203) to gain access to the database. Data extraction was performed using Structured Query Language (SQL) via Postgres SQL software (version 13.9) and Navicat Premium software (version 16.3). Study design followed STROBE reporting standards for observational research.

### Study population

**Figure 1:**
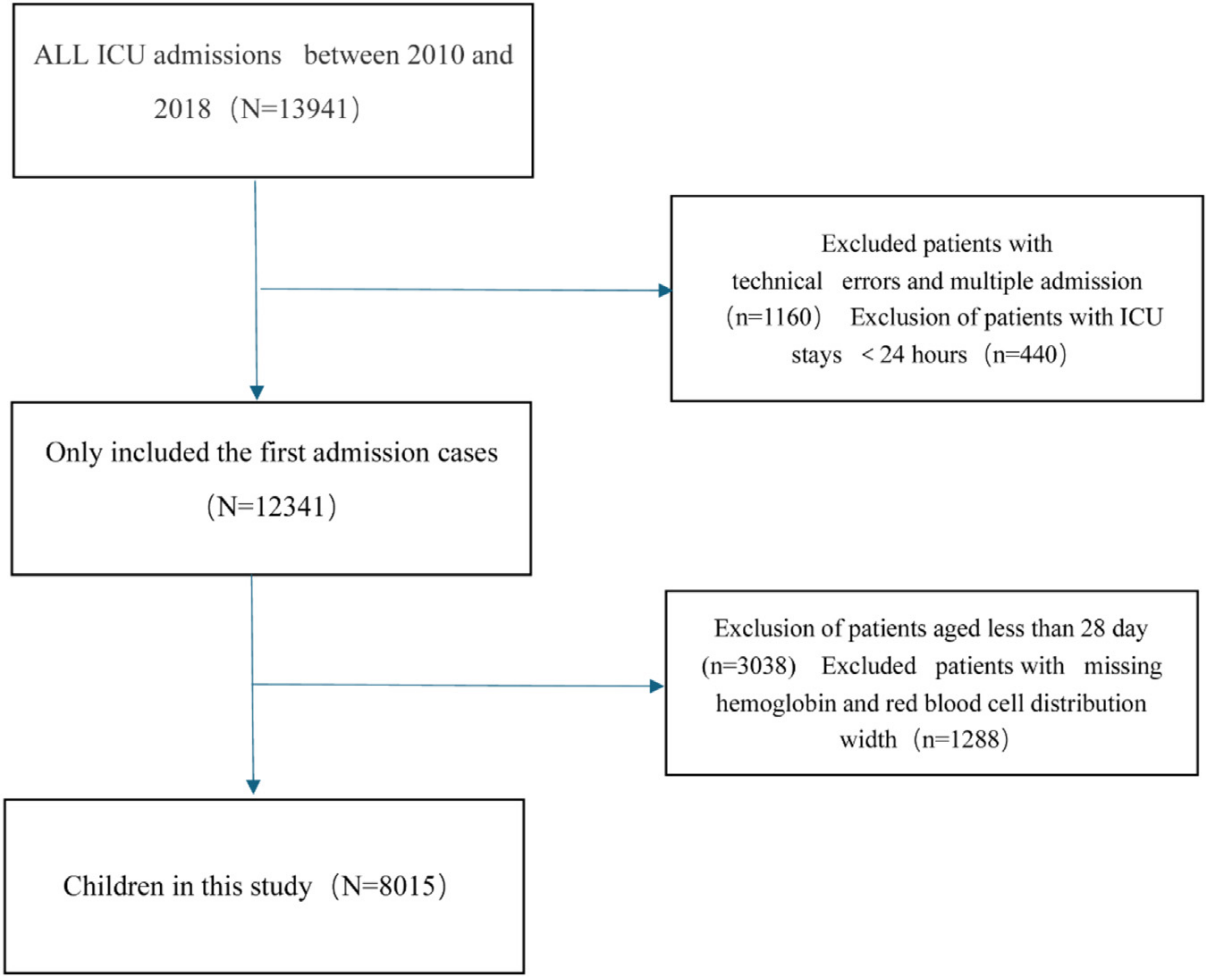
Flow chart of this cohort study population.

The cohort comprised 8,015 pediatric patients (28 days to < 18 years) admitted to non-neonatal ICUs. Initial hospitalization records were prioritized for analysis. Exclusion criteria included: 1) Missing hemoglobin/RDW measurements, 2) ICU duration < 24 hours. The flowchart of this cohort study is illustrated in Figure 1.

### Study variables and outcome

#### Exposure variable

HRR, calculated as Hb (g/L) divided by RDW (%), was measured within the first 24 hours of admission. For multiple measurements, the first recorded values were used.

#### Variable

Based on previous literature and the clinical practice, the authors included the following variables, which are considered confounding factors for the prognosis of critically ill pediatric patients: Demographics (age, sex, race), ICU Unit category (CICU, GICU, PICU, SIC). Vital Signs (Temperature, Respiratory Rate, Heart Rate, Systolic Blood Pressure, Diastolic Blood Pressure, Oxygen saturation), Comorbidities (Sepsis, Pneumonia, Encephalitis, Congenital heart diseases, Shock, Malignant cancer). Laboratory Indicators (White blood cells, Neutrophils Count, Lymphocyte Count, Monocyte Count, Hemoglobin, Red blood cell distribution width, Platelet, Albumin, Alanine Aminotransferase (ALT), Aspartate Aminotransferase (AST), Bilirubin Total, Lactate Dehydrogenase, Blood urea nitrogen, Creatinine, Triglycerides, Cholesterol total, Glucose, Potassium, Sodium, Chloride, Lactate, C-reactive protein, Procalcitonin, Fibrinogen, Ferritin, D-dimer, Humanil6, ICU length of stay (LOS), Hospital length of stay (LOS), ICU mortality, Hospital mortality. All laboratory data were collected from critically ill pediatric patients within the first 24 hours of admission to the PICU.

#### Outcomes

The primary outcome was the 28-day in-hospital all-cause mortality. Defined as death occurring in the ICU after admission. Patients were followed until death, discharge from the hospital, or the 28th day after admission, whichever occurred first.

### Statistical analysis

Categorical variables are expressed as frequencies and percentages, whereas continuous variables are presented as means with standard deviations (SDs) for normally distributed data or medians with interquartile ranges (IQRs) for skewed distributions. Group differences were assessed using the chi-squared test and Fisher’s exact test for categorical variables, and the Student’s t-test or Mann–Whitney U test for continuous variables.

To explore the independent associations between HRR and 28-day in-hospital all-cause mortality following admission to the PICU, the authors analyzed the relationship between the HRR and mortality using univariate and multivariate Cox proportional hazards models, reporting hazard ratios (HRs) along with their 95% confidence intervals (95% CIs). Patients were categorized into four groups based on serum HRR levels: Q1 (< 6.51), Q2 (6.52–7.78), Q3 (7.79–9.04), and Q4 (≥ 9.05), with Q1 serving as the reference group. Multivariate Cox regression analysis incorporated variables with a significance level of p < 0.05 from the univariate model. Three models were constructed to assess the independent association between HRR and 28-day in-hospital all-cause mortality: **Model Ⅰ**: Adjusted for age, sex, and ethnicity. **Model Ⅱ**: Additionally adjusted for ICU unit, sepsis, pneumonia, encephalitis, shock, and malignant cancer. **Model Ⅲ**: Further adjusted for temperature, respiratory rate, heart rate, systolic pressure, oxygen saturation, white blood cells, lymphocyte, platelet count, alanine aminotransferase, aspartate aminotransferase, bilirubin total, lactated hydrogenase, glucose, sodium, potassium, chloride, blood urea nitrogen, creatinine, lactate, C-reactive protein, procalcitonin, and fibrinogen.

In addition, A restricted cubic spline (RCS) regression was performed using four knots at the 5th, 35th, 65th, and 95th percentiles of HRR to assess linearity and examine the dose–response relationship between HRR and all-cause mortality, after adjusting for variables in **Model Ⅲ**. A two-piecewise logistic regression model with a smoothing curve was employed to identify the association threshold between HRR and all-cause mortality, with inflection points determined through likelihood-ratio tests and bootstrap resampling. Kaplan–Meier survival curves were generated to compare survival probabilities across HRR quintiles.

Furthermore, the authors also conducted a stratified analysis to investigate factors that may influence the association between HRR and 28-day all-cause mortality. Stratified and interaction analyses were performed based on age categories (< 1 year, 1 ≤ age < 3 years, and ≥ 3 years), sex (male or female), pneumonia (yes or no), encephalitis (yes or no), malignant cancer (yes or no), white blood cell count (< 10^9/L or ≥ 10^9/L), glucose levels (< 7 or ≥ 7 mmol/L), and lactate levels (< 2 or ≥ 2 mmol/L). Each stratification was adjusted for confounders in **Model Ⅱ**, excluding the stratification factor itself. The authors employed an interaction test within the Cox regression model to compare hazard ratios (HRs) across the analyzed subgroups.

To avoid bias, the authors excluded variables with over 30% missing values, including interleukin-6, ferritin, D-dimer, and total calcium from the analysis. Continuous variables with missing data under 10% were imputed using multiple imputation methods. To evaluate the robustness of the present findings, sensitivity analyses were conducted by excluding extremely high values of HRR (HRR > 12) and removing participants with congenital heart disease. Additionally, the authors excluded patients whose intensive care unit (ICU) stays were shorter than 48 hours and those with stays of less than 72 hours.

Given that the determination of the sample size was exclusively reliant on the provided data, no a priori statistical power estimates were conducted. All the analyses were performed using the statistical software packages R (http://www.R-project.org, The R Foundation) and Free Statistics software versions 2.2. A two-tailed test was performed and p < 0.05 was declared statistically significant.

## Results

### Baseline characteristics of population

Table 1 illustrates the baseline characteristics of the 8015 participants who were divided into four groups based on serum HRR level: Q1(< 6.51);Q2 (6.52-7.78);Q3 (7.79-9.04);Q4(≥9.05). The average age of the study participants was 1.5 years (0.4 to 4.8) and 3547 (44.3%) individuals were female. Generally, the median length of stay in the ICU was 1.9 days (0.9 to 5.5), while the median length of stay in the hospital was 12.0 days (7.1 to 18.8). The overall in-hospital all-cause mortality rate was 4.8% (385 patients), and the 28-day in-hospital all-cause mortality rate was 4.1% (330 patients). Mortality rates across the HRR quartiles were 6.1% (122 patients) for Q1, 3.5% (69 patients) for Q2, 2.7% (54 patients) for Q3, and 4.2% (85 patients) for Q4 (***p*** < 0.0001). Participants in the highest quartile of the HRR (Q4 ≥ 9.05) were primarily female and older individuals of Han ethnicity/race. Notably, they exhibited significantly higher levels of systolic pressure, diastolic pressure, white blood cells, neutrophils, hemoglobin, platelet count, albumin, lactate, and fibrinogen. Lower levels of respiratory rate, heart rate, lymphocyte, RDW, alanine aminotransferase, aspartate aminotransferase, lactate dehydrogenase, glucose, potassium, C-reactive protein, procalcitonin, human-IL6, D-dimer, and both ICU and hospital length of stay, compared to individuals in the lowest HRR quartile (Q1 < 6.51) (all p < 0.05). Additionally, the high HRR group showed a significantly higher prevalence of encephalitis, whereas sepsis, pneumonia, congenital heart disease, and malignant cancer were less prevalent in this group.

**Table 1:** Baseline characteristics of critically ill pediatric patients from Pediatric Intensive Care Unit database 2010-2018 by categories of HRR levels.

Variables	Total	hemoglobin to red blood cell distribution width ratio(%)				*P-*value
		Q1≤6.51	Q2 (6.52-7.78)	Q3 (7.79-9.04)	Q4≥9.05	
Number	8015	1988	1997	2016	2014	
Age, (year)	1.5 (0.4, 4.8)	0.8 (0.3, 2.6)	0.8 (0.3, 2.5)	1.6 (0.5, 4.1)	4.8 (1.8, 9.0)	< 0.001
Sex, n (%)						0.035
Female	3547 (44.3)	837 (42.1)	901 (45.1)	935 (46.4)	874 (43.4)	
Male	4468 (55.7)	1151 (57.9)	1096 (54.9)	1081 (53.6)	1140 (56.6)	
Ethnicity, n (%)						< 0.001
Others*	73 (0.9)	34 (1.7)	13 (0.7)	17 (0.8)	9 (0.4)	
Han	7942 (99.1)	1954 (98.3)	1984 (99.3)	1999 (99.2)	2005 (99.6)	
ICU. unit, n (%)						< 0.001
GICU	1730 (21.6)	440 (22.1)	301 (15.1)	401 (19.9)	588 (29.2)	
PICU	1648 (20.6)	538 (27.1)	360 (18)	360 (17.9)	390 (19.4)	
SICU	2377 (29.7)	517 (26)	739 (37)	640 (31.7)	481 (23.9)	
CICU	2260 (28.2)	493 (24.8)	597 (29.9)	615 (30.5)	555 (27.6)	
Sepsis, n (%)						< 0.001
No	7904 (98.6)	1936 (97.4)	1966 (98.4)	1995 (99)	2007 (99.7)	
Yes	111 (1.4)	52 (2.6)	31 (1.6)	21 (1)	7 (0.3)	
Pneumonia, n (%)						< 0.001
No	7340 (91.6)	1757 (88.4)	1800 (90.1)	1872 (92.9)	1911 (94.9)	
Yes	675 (8.4)	231 (11.6)	197 (9.9)	144 (7.1)	103 (5.1)	
Encephalitis, n (%)						< 0.001
No	7780 (97.1)	1947 (97.9)	1960 (98.1)	1962 (97.3)	1911 (94.9)	
Yes	235 (2.9)	41 (2.1)	37 (1.9)	54 (2.7)	103 (5.1)	
Shock, n (%)						0.198
No	8000 (99.8)	1985 (99.8)	1996 (99.9)	2012 (99.8)	2007 (99.7)	
Yes	15 (0.2)	3 (0.2)	1 (0.1)	4 (0.2)	7 (0.3)	
Congenital heart diseases, n (%)						< 0.001
No	7579 (94.6)	1864 (93.8)	1843 (92.3)	1921 (95.3)	1951 (96.9)	
Yes	436 (5.4)	124 (6.2)	154 (7.7)	95 (4.7)	63 (3.1)	
Malignant cancer, n (%)						< 0.001
No	7331 (91.5)	1747 (87.9)	1858 (93)	1872 (92.9)	1854 (92.1)	
Yes	684 (8.5)	241 (12.1)	139 (7)	144 (7.1)	160 (7.9)	
Temperature, (°C)	36.8 ± 0.9	36.9 ± 0.8	36.8 ± 0.8	36.8 ± 0.8	36.8 ± 1.1	0.26
Respiratory rate, (bpm)	29.3 ± 9.9	31.5 ± 14.2	30.1 ± 7.7	29.0 ± 8.1	26.3 ± 7.4	< 0.001
Heart rate, (bpm)	126.7 ± 26.2	133.7 ± 24.5	131.7 ± 23.0	125.3 ± 25.2	114.5 ± 28.3	< 0.001
Systolic pressure, (mmHg)	104.3 ± 26.3	100.8 ± 16.1	102.8 ± 29.1	103.9 ± 14.5	110.3 ± 38.2	< 0.001
Diastolic pressure, (mmHg)	61.4 ± 15.4	58.7 ± 14.3	60.0 ± 15.6	61.5 ± 12.8	65.9 ± 17.9	< 0.001
Oxygen saturation, (%)	98.5 ± 2.9	98.3 ± 4.2	98.6 ± 2.2	98.6 ± 2.5	98.6 ± 2.8	0.02
White blood cells, (10^9/L)	9.3 (6.4, 13.3)	8.5 (5.5, 13.1)	8.7 (6.1, 12.9)	9.3 (6.6, 13.0)	10.5 (7.7, 14.2)	< 0.001
Neutrophils, (10^9/L)	6.1 (3.6, 9.7)	5.0 (2.7, 8.6)	5.4 (3.1, 9.0)	6.1 (3.8, 9.5)	7.7 (4.9, 11.2)	< 0.001
Lymphocyte, (10^9/L)	2.2 (1.4, 3.2)	2.2 (1.4, 3.5)	2.3 (1.5, 3.3)	2.3 (1.5, 3.2)	2.0 (1.3, 2.9)	< 0.001
Monocyte, (10^9/L)	0.3 (0.2, 0.5)	0.3 (0.2, 0.6)	0.3 (0.2, 0.5)	0.3 (0.2, 0.5)	0.3 (0.2, 0.5)	< 0.001
Hemoglobin, (10^9/L)	107.0 ± 20.3	85.5 ± 16.7	101.4 ± 10.1	111.9 ± 9.3	128.8 ± 14.0	< 0.001
Platelet, (10^9/L),	280.6 ± 147.4	257.8 ± 180.6	278.6 ± 147.6	291.2 ± 134.8	294.7 ± 117.2	< 0.001
Red blood cell distribution width, (%)	14.1 ± 2.3	16.4 ± 3.2	14.1 ± 1.4	13.4 ± 1.1	12.7 ± 1.0	< 0.001
Alanine aminotransferase, (U/L)	20.0 (13.0, 34.0)	23.0 (14.0, 42.2)	21.0 (14.0, 32.0)	20.0 (13.0, 33.0)	18.0 (11.0, 31.0)	< 0.001
Aspartate aminotransferase, (U/L)	46.0 (30.0, 85.0)	59.5 (33.0, 113.0)	54.0 (34.0, 90.0)	43.0 (30.0, 78.0)	34.0 (25.0, 56.0)	< 0.001
Albumin, (g/L)	37.3 ± 5.9	35.3 ± 6.3	37.1 ± 5.5	37.9 ± 5.2	39.3 ± 5.6	< 0.001
Lactated hydrogenase, (U/L)	371.0 (272.0, 533.0)	437.5 (311.0, 629.8)	400.0 (301.0, 545.0)	348.0 (264.0, 490.0)	299.0 (234.0, 432.0)	< 0.001
Bilirubin total, (µmol/L)	9.8 (6.1, 16.5)	11.3 (6.5, 21.6)	10.4 (6.3, 17.4)	9.0 (5.8, 14.5)	9.1 (6.0, 13.5)	< 0.001
Creatinine, (µmol/L)	53.7 ± 314.4	57.9 ± 217.7	43.6 ± 86.1	52.1 ± 377.6	61.9 ± 459.5	0.339
Blood urea nitrogen, (mmol/L)	3.9 ± 3.8	4.4 ± 5.5	3.6 ± 3.5	3.6 ± 2.6	3.9 ± 2.5	< 0.001
Glucose, (mmol/L)	8.2 ± 3.8	8.3 ± 4.0	8.4 ± 3.7	8.0 ± 3.6	7.9 ± 3.7	< 0.001
Sodium, (mmol/L)	137.8 ± 5.2	137.7 ± 5.8	138.0 ± 5.0	137.7 ± 4.7	137.6 ± 5.1	0.196
Potassium, (mmol/L)	3.7 ± 0.7	3.7 ± 0.7	3.7 ± 0.7	3.7 ± 0.7	3.6 ± 0.8	< 0.001
Chloride, (mmol/L)	108.5 ± 5.9	107.9 ± 6.3	108.4 ± 5.7	109.1 ± 5.5	108.6 ± 5.8	< 0.001
Lactate, (mmol/L)	1.7 (1.2, 2.6)	1.7 (1.2, 2.7)	1.7 (1.2, 2.5)	1.6 (1.1, 2.4)	1.8 (1.3, 2.7)	< 0.001
Calcium total, (mmol/L)	2.3 ± 0.2	2.2 ± 0.2	2.3 ± 0.2	2.3 ± 0.2	2.3 ± 0.2	< 0.001
C-reactive protein, (mg/dl)	15.0 (4.0, 47.0)	20.0 (5.0, 52.0)	23.0 (5.0, 53.0)	13.0 (4.0, 43.3)	7.0 (4.0, 31.4)	< 0.001
Procalcitonin, (ng/ml)	0.4 (0.1, 1.7)	0.6 (0.2, 2.4)	0.4 (0.1, 1.5)	0.3 (0.1, 1.6)	0.2 (0.1, 1.2)	< 0.001
Human il6, (ng/L)	27.4 (8.5, 91.1)	37.5 (11.3, 109.4)	25.2 (8.3, 89.5)	24.8 (8.6, 86.6)	21.4 (6.4, 75.4)	< 0.001
Fibrinogen, (g/L),	2.3 ± 1.1	2.2 ± 1.1	2.3 ± 1.0	2.3 ± 1.0	2.4 ± 1.0	< 0.001
Ferritin, (ug/L)	52.2 (32.1, 103.5)	56.2 (27.8, 143.8)	48.0 (32.5, 86.9)	51.1 (33.6, 90.9)	56.3 (36.5, 101.1)	0.036
D-dimer, (g/L),	1.3 (0.9, 2.2)	1.5 (0.9, 2.3)	1.3 (0.9, 2.0)	1.3 (0.8, 2.1)	1.1 (0.7, 2.1)	< 0.001
**HRR, (g/L%)**	7.8 ± 1.9	5.3 ± 1.1	7.2 ± 0.4	8.4 ± 0.4	10.1 ± 0.9	< 0.001
Los, (days)	1.9 (0.9, 5.5)	3.0 (1.0, 7.1)	1.9 (0.9, 4.9)	1.1 (0.9, 4.4)	1.0 (0.8, 4.8)	< 0.001
Hospital. Los, (days)	12.0 (7.1, 18.8)	13.1 (7.3, 21.8)	13.1 (8.2, 19.6)	11.7 (7.2, 17.1)	10.1 (6.7, 16.6)	< 0.001
28 day-mortality, n (%)	330 (4.1)	122 (6.1)	69 (3.5)	54 (2.7)	85 (4.2)	< 0.001
ICU mortality, n (%)	387 (4.8)	140 (7)	80 (4)	72 (3.6)	95 (4.7)	< 0.001
Hospital mortality, n (%)	385 (4.8)	139 (7)	79 (4)	72 (3.6)	95 (4.7)	< 0.001

Data are shown as the mean ± SD or median (IQR) for skewed variables or numbers (proportions) for categorical variables.

**Note**:Others* ethnic groups include the Hui ethnic, baiyue ethnic, miao ethnic, tujia ethnic, yi ethnic, other ethnic;

HRR, hemoglobin to red blood cell distribution width ratio; Q, Quintile; CICU, cardiac intensive care unit; GICU, general intensive care unit; NICU, neonatal intensive care unit; PICU, pediatric intensive care unit; SICU, surgical intensive care unit. LOS, The length of stay for the patient for the given ICU stay. Hospital LOS: The length of stay for the patient for the given hospital stay.

### Association between HRR and 28 d in-hospital all-cause mortality

Cox regression analysis was conducted to identify factors associated with 28-day in-hospital all-cause mortality. The results of the univariate regression analysis indicated that sex, ethnicity, ICU unit, temperature, respiratory rate, heart rate, systolic blood pressure, oxygen saturation, sepsis, pneumonia, encephalitis, shock, hemoglobin, lymphocytes, platelet count, red blood cell distribution width, albumin, alanine aminotransferase, aspartate aminotransferase, lactate dehydrogenase, total bilirubin, creatinine, potassium, chloride, lactate, C-reactive protein, procalcitonin, fibrinogen, human IL-6, D-dimer, and hospital length of stay were positively associated with mortality (all p < 0.05) (Supplementary Table 1).

Table 2 presents the association between HRR and all-cause mortality in a multiple Cox regression model. When HRR levels were analyzed using quartiles, the fully adjusted Model Ⅲ indicated that, using Q3 (7.79 ≤ HRR < 9.04) as the reference group, the adjusted hazard ratios (HR) (95% CI) for all-cause mortality in Q1 (< 6.51), Q2 (6.52-7.78), and Q4 (≥ 9.05) were 1.22 (0.86–1.73), 1.10 (0.76–1.60), and 1.64 (1.14–2.36), respectively (*P* for trend = 0.22). The association between HRR and in-hospital mortality among pediatric patients remained stable across different subgroups after adjusting for potential confounders, with the exception of lactate (***p*** for interaction > 0.05).

**Table 2:** Association between HRR and 28-day all-cause mortality.

Variable	Non-adjusted	*P* value	Model I	*P* value	Model II	*P* value	Model III	*P* value
	HR (95%CI)		HR (95%CI)		HR (95%CI)		HR (95%CI)	
HRR, Quintile								
Q1(<6.51)	2.02 (1.46∼2.78)	<0.001	1.96 (1.42∼2.7)	<0.001	1.77 (1.28∼2.45)	0.001	1.22 (0.86∼1.73)	0.269
Q2(6.52-7.78)	1.17 (0.82∼1.67)	0.391	1.16 (0.81∼1.66)	0.418	1.24 (0.86∼1.77)	0.248	1.1 (0.76∼1.6)	0.618
Q3(7.79-9.04)	1(Ref)		1(Ref)		1(Ref)		1(Ref)	
Q4(>9.05)	1.71 (1.22∼2.41)	0.002	1.8 (1.27∼2.56)	0.001	1.6 (1.12∼2.27)	0.009	1.64 (1.14∼2.36)	0.008
*P* for Trend		0.083		0.139		0.144		0.22
								

HRR, hemoglobin to red blood cell distribution width ratio; Q, quartiles; HR, hazard ratio; CI, confidence interval; Ref, reference.

**Model I**: adjusted by age +sex +ethnicity;

**Model II**: adjusted by **Model I** +ICU unit +sepsis +pneumonia +encephalitis +shock +malignant cancer;

**Model III**: adjusted by **Model II** +temperature +respiratory rate +heartrate +systolic pressure +oxygen saturation +white blood cells +lymphocyte +platelet + albumin +alanine aminotransferase+ aspartate aminotransferase+ bilirubin total +glucose +sodium +potassium + chloride + blood urea nitrogen +creatinine + lactate+ C-reactive protein + procalcitonin + fibrinogen.

**Figure 2:**
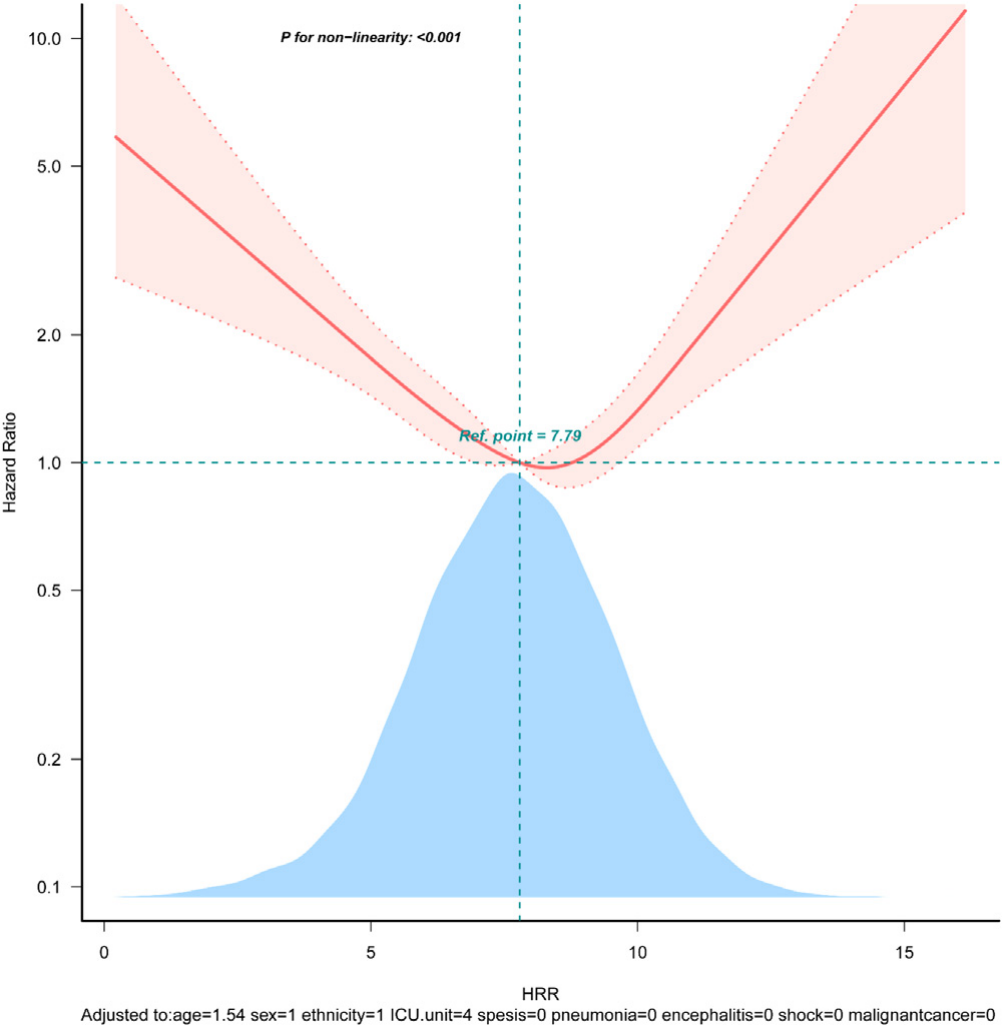
Restricted cubic spline plot for HRR and 28-day all-cause mortality. The restricted cubic spline depicts the hazard ratio of the hemoglobin to albumin ratio associated with all-cause mortality among critically ill pediatric patients. The x-axis represents the HRR, while the y-axis depicts the hazard ratio of all-cause mortality. The model was adjusted for age, sex, ethnicity, ICU unit, sepsis, pneumonia, encephalitis, shock, malignant cancer, temperature, respiratory rate, heart rate, systolic pressure, oxygen saturation, white blood cells, lymphocyte, platelet count, alanine aminotransferase, aspartate aminotransferase, bilirubin total, lactated hydrogenase, glucose, sodium, potassium, chloride, blood urea nitrogen, creatinine, lactate, C-reactive protein, procalcitonin, fibrinogen. Solid and dashed lines represent the predicted value and 95% confidence intervals. Abbreviations: HRR: hemoglobin to red blood cell distribution width ratio.

Restricted cubic spline analysis accurately described a U-shaped association between HRR and the 28-day all-cause mortality (*p* for nonlinearity < 0.05, Figure 2) after adjusting for covariates in Model Ⅲ. The nadir of risk was identified at an HRR of 8.91, as determined through a two-piecewise linear regression model. Notably, to the left of the inflection point, the risk of mortality decreased with increasing levels of the HRR (HR: 0.803, 95% CI: 0.742∼0.869, p < 0.001). Conversely, an inverse relationship was observed to the right of the inflection point (HR:1.421, 95% CI:1.159 ∼1.743, p < 0.001) (Table 3).

**Table 3:** Threshold effect analysis for the relationship between HRR and all-cause mortality.

HRR	HR	95%CI	*P*-value
Turning point (%)	8.91	8.744-9.075	
HRR<8.91	0.803	0.742-0.869	< 0.001
HRR ≥8.91	1.421	1.159-1.743	< 0.001
Likelihood Ratio test			<0.001

HRR, hemoglobin to red blood cell distribution width ratio; Q, quartiles; HR, hazard ratio; CI, confidence interval. They were adjusted for age, sex, ethnicity, ICU unit, sepsis, pneumonia, encephalitis, shock, malignant and cancer.

### Kaplan–Meier survival analysis

**Figure 3:**
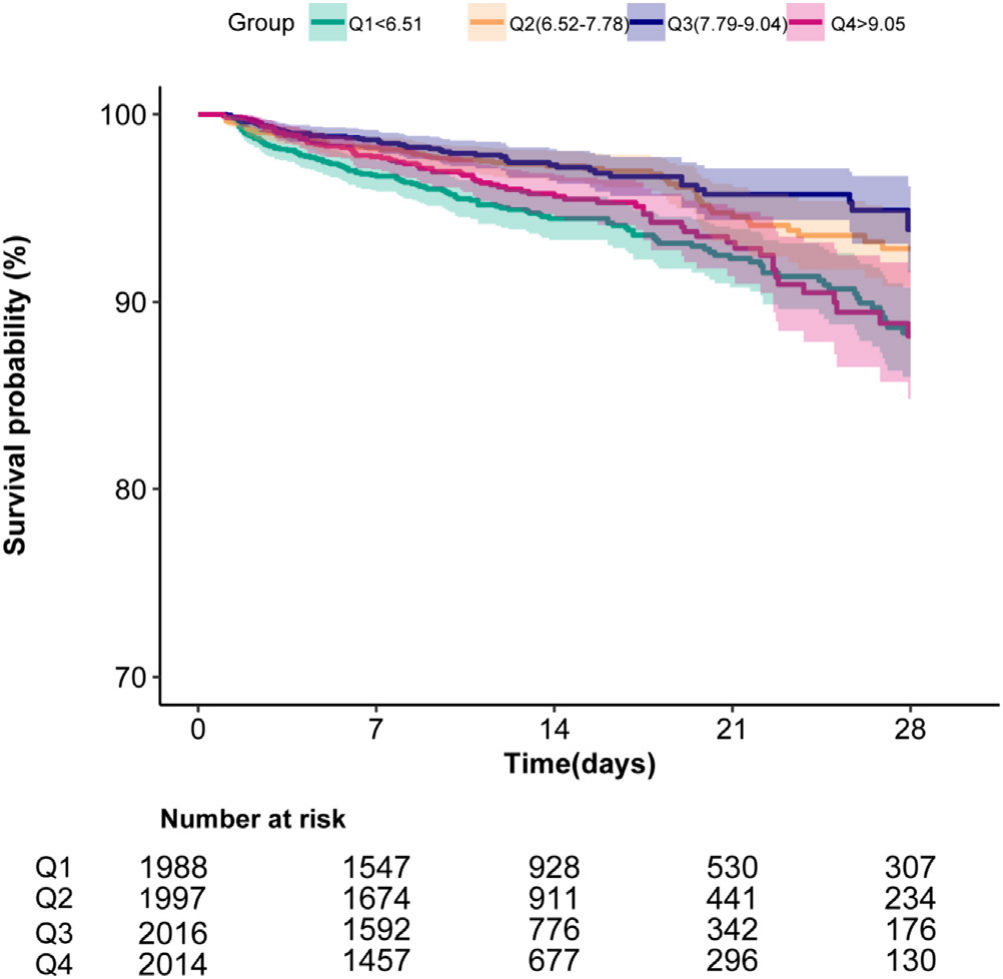
Kaplan–Meier curve of 28-day all-cause mortality for critically ill pediatrics. The curved line and shaded areas depict the estimated values and their corresponding 95% confidence intervals. Only patients with a hospital length of stay ≤ 28 days are displayed.

To evaluate the cumulative survival period at different HRR groups, Kaplan–Meier survival curves were generated based on HRR quartiles. The curves demonstrated significantly higher 28-day survival rates among patients with Q3 levels (7.79 ≤ HRR<9.04) compared to those in both the higher and lower HRR groups (*p* < 0.0001; Figure 3).

### Subgroup analyses

The authors conducted subgroup and interaction analyses while adjusting for confounders (**Model Ⅲ**). Subgroup analyses were performed by stratifying the data based on different variables, including age, sex, pneumonia, encephalitis, malignant cancer, white blood cells, glucose, and lactate. The authors found a significant interaction between lactate and HRR concerning all-cause mortality (***p*** for interaction < 0.05). However, no significant interactions were observed in other subgroups (*p* for interaction > 0.05). Detailed information regarding the association between HRR and all-cause mortality can be found in Figure S1.

### Sensitivity analysis

To ensure the robustness of the present study, the authors conducted several sensitivity analyses. First, the authors excluded extremely high values of HRR (HRR > 12) and still identified a U-shaped relationship between HRR and all-cause mortality after adjusting for potential confounders. Second, the authors excluded 436 patients with congenital heart disease, and the association remained robust. Third, this relationship persisted when the authors excluded patients with ICU stays of less than 48 hours (n = 206). Furthermore, after excluding patients with ICU stays of less than 72 hours (n = 433), the association continued to be significant in this cohort. Overall, the U-shaped association between HRR and all-cause mortality remained reliable (Supplementary Tables 2-5), indicating that both lower and higher HRR levels are associated with an increased risk of mortality.

## Discussion

This retrospective cohort study, utilizing the pediatric-specific PIC database in China, represents the first investigation into the relationship between the HRR and short-term mortality in critically ill pediatric patients. The present study revealed a U-shaped association between HRR and 28-day all-cause in-hospital mortality, with an inflection point at an HRR of 8.91. Subgroup analyses demonstrated that this association remained consistent across various patient groups, with notably pronounced effects in patients with elevated lactate levels. Both low and high HRR levels were linked to increased mortality risk. These findings suggest that maintaining HRR within a specific range may be crucial for mortality risk assessment in critically ill pediatric populations.

The emerging evidence has demonstrated the prognostic value of the HRR in various adult populations. Notably, Wang et al. reported that low HRR levels (HRR < 5.877) were associated with a 1.412-fold increase in 28-day all-cause and cardiac mortality,[24] while Lai et al. linked lower HRR to heightened risks of all-cause, cancer, and cardiovascular mortality.[25] Liu et al. reported non-linear associations of HRR with mortality in subarachnoid hemorrhage.[26] Furthermore, Liu et al. highlighted the strong link between lower HRR and both short-term and long-term mortality in critically ill patients with heart failure and acute kidney injury (AKI).[27] Chen et al. emphasized low HRR’s role in identifying hemophagocytic lymphohistiocytosis patients at risk for AKI and 28-day mortality.[28] While prior studies have excluded patients with elevated lactate levels, a key marker of poor prognosis in critically ill patients, the real-world clinical investigation included lactate indicators and incorporated pediatric patients with high lactate levels. Importantly, Consistent with previous research linking lower HRR levels to increased all-cause mortality, the present study uniquely identifies a U-shaped relationship between HRR and mortality, with a critical inflection point at approximately 8.91. This novel finding may be attributed to differences in study populations, observation periods, admission and discharge criteria, end-of-life decision-making, and treatment protocols. Pediatric ICU patients often present with distinct comorbidities along with varying disease durations and complications when compared to adult cohorts. These findings have significant implications for current management strategies aimed at mitigating all-cause mortality in critically ill children, suggesting that both low and high HRR levels require vigilant monitoring and intervention. Given these complexities, these results highlight the importance of maintaining an optimal HRR level within the pediatric population. The present study enriches the existing literature, emphasizing the necessity for personalized approaches in pediatric critical care.

The correlation mechanism between the HRR and mortality remains unclear. The present findings indicate that the U-shaped association may be influenced by underlying inflammatory and nutritional mechanisms. Lower HRR values may reflect anemia and elevated RDW, which are associated with inflammation and oxidative stress.[29]

Anemia impairs oxygen delivery, causing hypoxia and organ dysfunction, while inflammation disrupts iron metabolism and erythropoietin secretion, further reducing hemoglobin concentration.[30] Elevated RDW indicates immature red blood cells from cytokine-mediated suppression of erythropoiesis, creating a cycle of anemia and inflammation that worsens systemic damage.[3] These interrelated processes create a vicious cycle: inflammation drives anemia and dysregulated erythropoiesis, which in turn perpetuates systemic inflammation and organ injury. Notably, Higher HRR values also correlate with increased mortality, possibly due to hemoconcentration or reduced RDW deformability, leading to thrombotic risks or poor tissue perfusion. Chronic inflammation may modify HRR through hemoglobin’s anti-inflammatory effects and RDW reduction, mitigating tissue damage.[24] Genetic factors linked to RDW, such as telomere length and apoptosis, require further exploration.[31] A deeper understanding of the biological pathways linking RDW to various health outcomes may aid in identifying potential therapeutic targets.[32] Both high and low HRR levels necessitate close monitoring and intervention. Ultimately, HRR provides a comprehensive perspective on RDW and oxygen-carrying capacity, demonstrating its potential utility as a prognostic assessment tool in critically ill pediatric populations.

The present findings highlight the potential of the HRR as a novel integrative biomarker in pediatric critical care. Unlike isolated measurements of hemoglobin or RDW, HRR effectively captures the dynamic interplay between oxygen-carrying capacity and inflammatory-oxidative stress, thereby providing a more comprehensive prognostic framework. The identified U-shaped association underscores the necessity for personalized thresholds in clinical practice, as both low and high extremes of HRR are linked to adverse outcomes. Clinicians should consider implementing HRR-guided strategies to optimize anemia management, including targeted erythropoietin therapy, iron supplementation, and transfusion protocols, while also addressing underlying inflammatory processes. Furthermore, monitoring HRR levels may assist in identifying high-risk pediatric patients, and therapeutic approaches aimed at modulating hemoglobin and RDW could potentially improve patient outcomes.

However, this study has several limitations that warrant consideration. First, its retrospective design may involve uncontrolled confounders, though multivariate regression, stratification, and sensitivity analyses were used to enhance robustness and minimize bias. Second, the authors did not account for dynamic changes in the HRR following treatment, which may influence mortality risk. Future research should include longitudinal measurements to better elucidate the predictive significance of these biomarkers. Third, the single-center nature of this cohort study may limit the generalizability of these findings to broader populations. Nonetheless, the PIC database provides valuable insights into pediatric intensive care unit (ICU) data and complements the limited scope of MIMIC-III by focusing on critically ill children. Given these limitations, it is essential to design multicenter randomized controlled trials to further investigate the association between HRR levels and all-cause mortality.

## Conclusion

In conclusion, the present study reveals a U-shaped relationship between the HRR and all-cause mortality in critically ill pediatric patients, with optimal HRR levels around 8.91 associated with reduced risk.

## Institutional review board statement

The present study was approved by the Institutional Review Board/Ethical Committee of the Children's Hospital, Zhejiang University School of Medicine (Hangzhou, China 2019_IRB_052). Ethical review and approval were waived for this study because no additional institutional review board approval was required for the secondary analysis. Given the retrospective nature of the study and the utilization of anonymized patient data, the requirement for obtaining individual patient consent was waived. The Cangzhou Central Hospital institutional review board determined the study to be exempt because it used publicly available deidentified data, and informed consent was waived.

## Authors’ contributions

Weichao He wasesponsible for selecting topics, designing, and writing the original draft. Jie Liu contributed to conception, review, and editing of articles. Rui Jiang, Xinyu Yang, Zhenshui Hu contributed to the conception and design of the study, and analysis and interpretation of data. Xujie Zhang, Ruoyu Cao, Xiaolan Zhang, Yan Gao contributed to the conception and design of the study, and drafted the article. All authors have read and approved the final manuscript.

## Funding statement

This research was supported by the Medical Science Research Project of Hebei Province (20251533) and Liangduo Jiang Famous Traditional Chinese Medicine Inheritance Studio.

## Abbreviations

HRR: hemoglobin to red blood cell distribution width ratio; CICU: cardiac intensive care unit; GICU: general intensive care unit; NICU: neonatal intensive care unit; PICU: pediatric intensive care unit; SICU: surgical intensive care unit. LOS: The length of stay for the patient for the given ICU stay. Hospital LOS: The length of stay for the patient for the given hospital stay.

## Declarations

This article is polished with ChatGPT assistance.

## Data availability statement

More information regarding the data can be found on the PIC website (https://pic.nbscn.org/).

## Conflicts of interest

The authors declare no conflict of interest.

## Appendix Supplementary materials

Image, application 1
