# Supplementary material for: U-shaped association between hemoglobin to red blood cell distribution width ratio and all-cause mortality among critically ill pediatric patients
Source: J Pediatr (Rio J). 2025 Dec 17;102(1):101491. doi: 10.1016/j.jped.2025.101491 (PMC12774735; doi:10.1016/j.jped.2025.101491)
Supplement: Supplementary file 1 [file mmc1.docx]

**Supplementary Material**

**Table S1** Univariate analysis for all-cause mortality.

| Variable | HR (95%CI) | ***P*** value |
| --- | --- | --- |
| Age (year) | 0.9906 (0.9619,1.0201) | 0.526 |
| Sex (male), n (%) | 1.32 (1.06,1.65) | 0.014 |
| Ethnicity (Others), n (%) | 0.35 (0.17,0.75) | 0.007 |
| ICU unit: ref.=GICU |  |  |
| PICU | 0.83 (0.65,1.06) | 0.141 |
| CICU | 0.15 (0.1,0.21) | < 0.001 |
| SICU | 0.18 (0.12,0.26) | < 0.001 |
| Temperature (℃) | 0.79 (0.72,0.87) | < 0.001 |
| Respiratory rate (bpm) | 1.0076 (1.0045,1.0108) | < 0.001 |
| Heart rate (bpm) | 1.0077 (1.0017,1.0138) | 0.013 |
| Systolic blood pressure (mmHg) | 0.98 (0.97,0.99) | < 0.001 |
| Diastolic blood pressure (mmHg) | 0.9933 (0.9795,1.0072) | 0.344 |
| Oxygen saturation (%) | 0.96 (0.94,0.97) | < 0.001 |
| Sepsis | 4.69 (2.95,7.45) | < 0.001 |
| Pneumonia | 2.12 (1.6,2.8) | < 0.001 |
| Encephalitis | 2.67 (1.79,3.99) | < 0.001 |
| Shock | 6.05 (1.51,24.32) | 0.011 |
| Congenital heart disease | 1.27 (0.85,1.9) | 0.242 |
| Malignant cancer | 0.73 (0.49,1.1) | 0.13 |
| White blood cells (10^9/L) | 1.0013 (0.9979,1.0047) | 0.447 |
| Neutrophils (10^9/L) | 1.0018 (0.9828,1.0213) | 0.851 |
| Hemoglobin (g/L) | 1.09 (1.07,1.11) | < 0.001 |
| Lymphocyte (10^9/L) | 1.26 (1.17,1.35) | < 0.001 |
| monocyte (10^9/L) | 1.0013 (0.9979,1.0047) | 0.447 |
| Platelet (10^9/L) | 0.998 (0.9973,0.9988) | < 0.001 |
| Red blood cell distribution width (%) | 1.08 (1.04,1.12) | < 0.001 |
| Alanine aminotransferase (U/L) | 1.0003 (1.0002,1.0004) | < 0.001 |
| Aspartate aminotransferase (U/L) | 1.0002 (1.0001,1.0002) | < 0.001 |
| Albumin (g/L) | 0.95 (0.93,0.97) | < 0.001 |
| Lactated hydrogenase (U/L) | 1.0001 (1.0001,1.0002) | < 0.001 |
| Bilirubin total (μmol/L) | 1.0033 (1.002,1.0046) | < 0.001 |
| Creatinine (µmol/L) | 1.06 (1.05,1.07) | < 0.001 |
| Blood urea nitrogen (mmol/L) | 1.0001 (0.9998,1.0003) | 0.535 |
| Glucose (mmol/L) | 1.01 (0.98,1.04) | 0.398 |
| Sodium (mmol/L) | 0.98 (0.96,1) | 0.075 |
| Potassium (mmol/L) | 1.25 (1.17,1.33) | < 0.001 |
| Chloride (mmol/L) | 0.94 (0.93,0.96) | < 0.001 |
| Calcium total (mmol/L) | 2.35 (0.48,11.39) | 0.29 |
| Lactate (cont. var.) | 1.21 (1.19,1.24) | < 0.001 |
| C-reactive protein (mg/dl) | 0.9959 (0.9918,1) | 0.051 |
| Procalcitonin (ng/ml) | 1.004 (1.0028,1.0053) | < 0.001 |
| Fibrinogen (g/L) | 0.68 (0.59,0.79) | < 0.001 |
| Ferritin (ug/L) | 1.0008 (0.9997,1.0018) | 0.141 |
| humanil6 (pg/ml) | 1.0003 (1.0001,1.0005) | 0.008 |
| D-dimer (ug/L) | 1.1 (1.05,1.15) | < 0.001 |
| **HRR** | 0 (0,0.11) | 0.003 |
| ICU LOS (day) | 1.0023 (0.9977,1.0069) | 0.332 |
| Hospital LOS (day) | 0 (0,0) | < 0.001 |

**Note**：Others* ethnic groups include the Hui ethnic, baiyue ethnic, miao ethnic, tujia ethnic, yi ethnic, other ethnic；

HRR, hemoglobin to red blood cell distribution width ratio; CICU, cardiac intensive care unit; GICU, general intensive care unit; NICU, neonatal intensive care unit; PICU, pediatric intensive care unit; SICU, surgical intensive care unit. LOS, The length of stay for the patient for the given ICU stay.  Hospital LOS: The length of stay for the patient for the given hospital stay.

**Table S2** Association between HRR and 28-day all-cause mortality excluded 86 patients with extremely large values of HRR＞12.

| **Variable** | **Non-adjusted** | ***P* value** | **Model I** | ***P* value** | **Model II** | ***P* value** | **Model III** | ***P* value** |
| --- | --- | --- | --- | --- | --- | --- | --- | --- |
|  | **HR (95%CI)** |  | **HR (95%CI)** |  | **HR (95%CI)** |  | **HR (95%CI)** |  |
| HRR, Quintile |  |  |  |  |  |  |  |  |
| Q1(＜6.5) | 2.07 (1.5~2.87) | <0.001 | 2.02 (1.46~2.8) | <0.001 | 1.82 (1.31~2.53) | <0.001 | 1.09 (0.76~1.55) | 0.642 |
| Q2(6.51-7.76) | 1.2 (0.83~1.71) | 0.332 | 1.19 (0.83~1.7) | 0.357 | 1.25 (0.87~1.8) | 0.223 | 1 (0.69~1.44) | 0.982 |
| Q3(7.77-8.99) | 1(Ref) |  | 1(Ref) |  | 1(Ref) |  | 1(Ref) |  |
| Q4(＞9) | 1.62 (1.14~2.3) | 0.007 | 1.7 (1.18~2.43) | 0.004 | 1.51 (1.05~2.16) | 0.026 | 1.63 (1.13~2.35) | 0.009 |
| *P* for Trend |  | 0.02 |  | 0.039 |  | 0.045 |  | 0.053 |

HRR, hemoglobin to red blood cell distribution width ratio; Q, quartiles; HR, hazard ratio; CI, confidence interval; Ref, reference.

**Model I**: adjusted by age +sex +ethnicity;

**Model II**: adjusted by **Model I** +ICU unit +sepsis +pneumonia +encephalitis +shock +malignant cancer;

**Model III**: adjusted by **Model II** +temperature +respiratory rate +heartrate +systolic pressure +oxygen saturation +white blood cells +lymphocyte +platelet + albumin +alanine aminotransferase+ aspartate aminotransferase+ bilirubin total +glucose +sodium +potassium + chloride + blood urea nitrogen +creatinine + lactate+ C-reactive protein + procalcitonin + fibrinogen.**Table S3** Association between HRR and 28-day all-cause mortality excluded 436 patients with congenital heart disease.

| **Variable** | **Non-adjusted** | ***P* value** | **Model I** | ***P* value** | **Model II** | ***P* value** | **Model III** | ***P* value** |
| --- | --- | --- | --- | --- | --- | --- | --- | --- |
|  | **HR (95%CI)** |  | **HR (95%CI)** |  | **HR (95%CI)** |  | **HR (95%CI)** |  |
| HRR, Quintile |  |  |  |  |  |  |  |  |
| Q1(＜6.53) | 2.1 (1.5~2.94) | <0.001 | 2.06 (1.47~2.88) | <0.001 | 1.83 (1.3~2.57) | <0.001 | 1.31 (0.91~1.89) | 0.152 |
| Q2(6.54-7.82) | 1.2 (0.83~1.75) | 0.33 | 1.21 (0.83~1.75) | 0.324 | 1.27 (0.87~1.85) | 0.216 | 1.15 (0.78~1.71) | 0.484 |
| Q3(7.83-9.07) | 1(Ref) |  | 1(Ref) |  | 1(Ref) |  | 1(Ref) |  |
| Q4(＞9.08) | 1.64 (1.14~2.35) | 0.007 | 1.67 (1.15~2.42) | 0.007 | 1.49 (1.03~2.15) | 0.036 | 1.56 (1.06~2.29) | 0.025 |
| *P* for trend |  | 0.023 |  | 0.025 |  | 0.04 |  | 0.661 |

HRR, hemoglobin to red blood cell distribution width ratio; Q, quartiles; HR, hazard ratio; CI, confidence interval; Ref, reference.

**Model I**: adjusted by age +sex +ethnicity;

**Model II**: adjusted by **Model I** +ICU unit +sepsis +pneumonia +encephalitis +shock +malignant cancer;

**Model III**: adjusted by **Model II** +temperature +respiratory rate +heartrate +systolic pressure +oxygen saturation +white blood cells +lymphocyte +platelet + albumin +alanine aminotransferase+ aspartate aminotransferase+ bilirubin total +glucose +sodium +potassium + chloride + blood urea nitrogen +creatinine + lactate+ C-reactive protein + procalcitonin + fibrinogen.

**Table S4** Association between HRR and 28-day all-cause mortality excluded 206 patients with ICU stays <48 hours.

| **Variable** | **Non-adjusted** | ***P* value** | **Model I** | ***P* value** | **Model II** | ***P* value** | **Model III** | ***P* value** |
| --- | --- | --- | --- | --- | --- | --- | --- | --- |
|  | **HR (95%CI)** |  | **HR (95%CI)** |  | **HR (95%CI)** |  | **HR (95%CI)** |  |
| HRR, Quintile |  |  |  |  |  |  |  |  |
| Q1(＜6.53) | 1.9 (1.33~2.7) | <0.001 | 1.87 (1.31~2.66) | <0.001 | 1.67 (1.17~2.39) | 0.005 | 1.34 (0.92~1.95) | 0.125 |
| Q2(6.54-7.79) | 1.09 (0.74~1.62) | 0.663 | 1.09 (0.74~1.62) | 0.66 | 1.13 (0.76~1.68) | 0.543 | 1.01 (0.67~1.53) | 0.963 |
| Q3(7.8-9.04) | 1(Ref) |  | 1(Ref) |  | 1(Ref) |  | 1(Ref) |  |
| Q4(＞9.05) | 1.94 (1.35~2.79) | <0.001 | 1.97 (1.35~2.86) | <0.001 | 1.75 (1.2~2.54) | 0.004 | 1.89 (1.28~2.79) | 0.001 |
| *P* for trend |  | 0.755 |  | 0.655 |  | 0.71 |  | 0.175 |

HRR, hemoglobin to red blood cell distribution width ratio; Q, quartiles; HR, hazard ratio; CI, confidence interval; Ref, reference.

**Model I**: adjusted by age +sex +ethnicity;

**Model II**: adjusted by **Model I** +ICU unit +sepsis +pneumonia +encephalitis +shock +malignant cancer;

**Model III**: adjusted by **Model II** +temperature +respiratory rate +heartrate +systolic pressure +oxygen saturation +white blood cells +lymphocyte +platelet + albumin +alanine aminotransferase+ aspartate aminotransferase+ bilirubin total +glucose +sodium +potassium + chloride + blood urea nitrogen +creatinine + lactate+ C-reactive protein + procalcitonin + fibrinogen.

**Table S5** Association between HRR and 28-day all-cause mortality excluded 433 patients with ICU stays <72 hours.

| **Variable** | **Non-adjusted** | ***P* value** | **Model I** | ***P* value** | **Model II** | ***P* value** | **Model III** | ***P* value** |
| --- | --- | --- | --- | --- | --- | --- | --- | --- |
|  | **HR (95%CI)** |  | **HR (95%CI)** |  | **HR (95%CI)** |  | **HR (95%CI)** |  |
| HRR, Quintile |  |  |  |  |  |  |  |  |
| Q1(＜6.54) | 1.88 (1.29~2.74) | 0.001 | 1.84 (1.26~2.69) | 0.002 | 1.67 (1.14~2.45) | 0.008 | 1.28 (0.86~1.92) | 0.227 |
| Q2(6.55-7.79) | 1.14 (0.75~1.73) | 0.547 | 1.14 (0.75~1.73) | 0.541 | 1.18 (0.77~1.79) | 0.452 | 1.02 (0.66~1.59) | 0.917 |
| Q3(8-9.04) | 1(Ref) |  | 1(Ref) |  | 1(Ref) |  | 1(Ref) |  |
| Q4(＞9.05) | 2.01 (1.36~2.97) | <0.001 | 2.05 (1.37~3.07) | <0.001 | 1.83 (1.22~2.74) | 0.003 | 1.77 (1.17~2.69) | 0.007 |
| *P* for Trend |  | 0.943 |  | 0.875 |  | 0.889 |  | 0.217 |

HRR, hemoglobin to red blood cell distribution width ratio; Q, quartiles; HR, hazard ratio; CI, confidence interval; Ref, reference.

**Model I**: adjusted by age +sex +ethnicity;

**Model II**: adjusted by **Model I** +ICU unit +sepsis +pneumonia +encephalitis +shock +malignant cancer;

**Model III**: adjusted by **Model II** +temperature +respiratory rate +heartrate +systolic pressure +oxygen saturation +white blood cells +lymphocyte +platelet + albumin +alanine aminotransferase+ aspartate aminotransferase+ bilirubin total +glucose +sodium +potassium + chloride + blood urea nitrogen +creatinine + lactate+ C-reactive protein + procalcitonin + fibrinogen.

**Figure S1** Forest plot for the relationship between HRR and 28-day all-cause mortality.

Squares indicate hazard ratios (HRs), with horizontal lines indicating 95%CIs. Adjustment factors included were adjusted for age, sex, ethnicity, ICU unit, sepsis, pneumonia, encephalitis, shock, and malignant cancer. Only 99% of the data is shown.

**
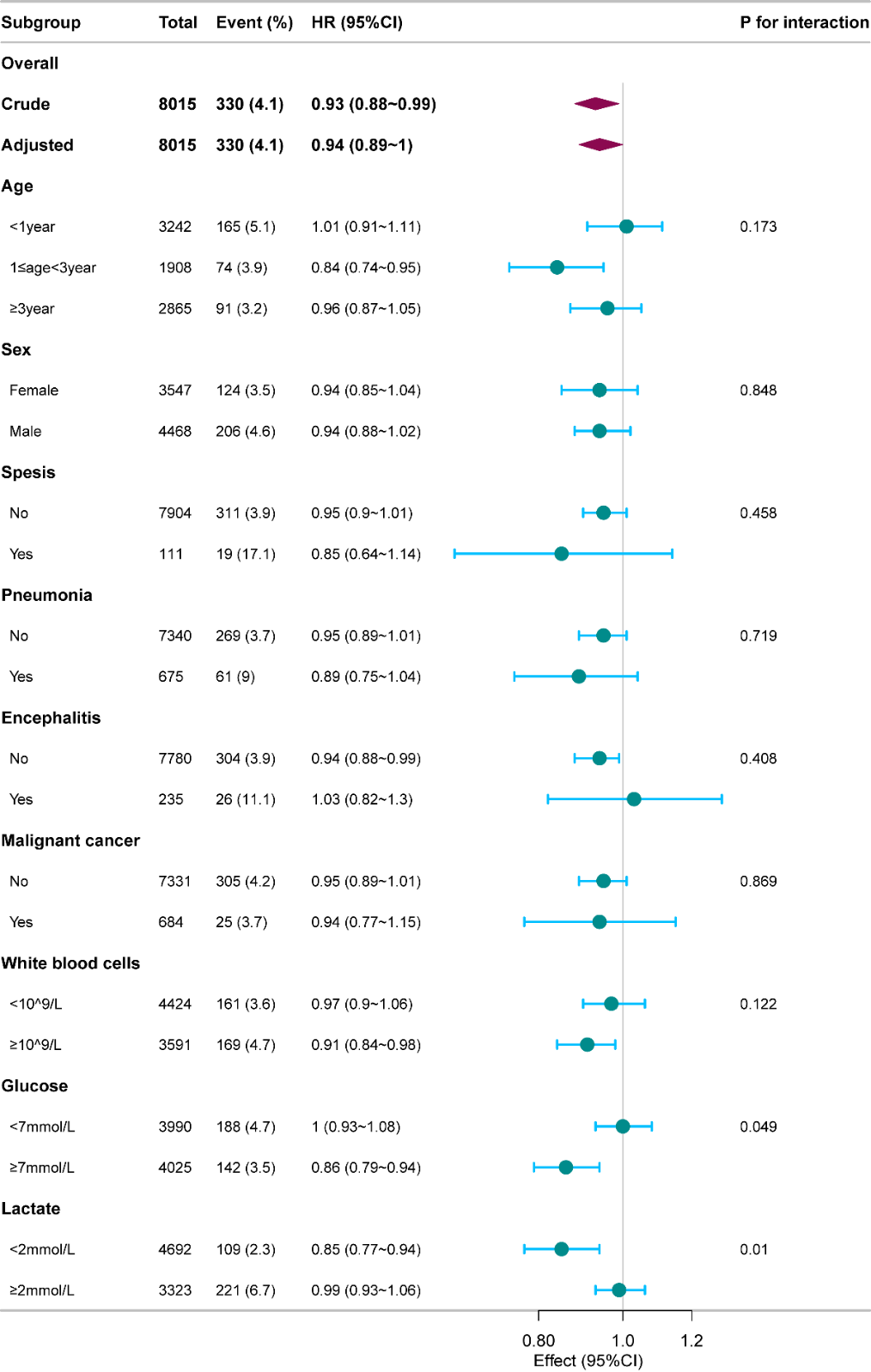
**
